# Effects of *FGFR4* G388R, V10I polymorphisms on the likelihood of cancer

**DOI:** 10.1038/s41598-020-80146-y

**Published:** 2021-01-14

**Authors:** Tao Peng, Yangyang Sun, Zhiwei Lv, Ze Zhang, Quanxin Su, Hao Wu, Wei Zhang, Wei Yuan, Li Zuo, Li Shi, Li-Feng Zhang, Xiaoli Zhou, Yuanyuan Mi

**Affiliations:** 1 Department of Urology, Affiliated Hospital of Jiangnan University, Wuxi, 214000 People’s Republic of China; 2 Department of Pathology, The Affiliated Changzhou No.2 People’s Hospital of Nanjing Medical University, 29 Xinglong Road, Changzhou, 213003 People’s Republic of China; 3 Department of Urology, The Affiliated Changzhou No.2 People’s Hospital of Nanjing Medical University, 29 Xinglong Road, Changzhou, 213003 People’s Republic of China; 4 Department of Oncology, Taizhou People’s Hospital, South Hailing Road 399, Taizhou, 225300 People’s Republic of China; 5 Department of Cardiology, Taizhou People’s Hospital, South Hailing Road 399, Taizhou, 225300 People’s Republic of China

**Keywords:** Genetic association study, Prostate

## Abstract

The correlation between G388R or V10I polymorphisms of *fibroblast growth factor receptor* (*FGFR*) 4 gene and the risk of carcinoma has been investigated previously, but the results are contradictory. Odds ratios (ORs) with 95% confidence intervals (95%CIs), in silico tools, and immunohistochemical staining (IHS) were adopted to assess the association. In total, 13,793 cancer patients and 16,179 controls were evaluated in our pooled analysis. Summarization of all the studies showed that G388R polymorphism is associated with elevated susceptibility to cancer under homozygous comparison (OR = 1.21, 95%CI = 1.03–1.43, *P* = 0.020) and a recessive genetic model (OR = 1.21, 95%CI = 1.04–1.41, *P* = 0.012). In the stratification analysis by cancer type and ethnicity, similar findings were indicated for prostate cancer, breast cancer, and individuals of Asian descendant. Polyphen2 bioinformatics analysis showed that the G388R mutation is predicted to damage the protein function of FGFR4. IHS analysis indicated that FGFR4 expression is increased in advanced prostate cancer. These findings may guide personalized treatment of certain types of cancers. Up-regulation of *FGFR4* may be related to a poor prognosis in prostate cancer.

## Introduction

Cancer remains a global threat to public health and poses a huge economic burden on the societies of both developing and developed countries. Breast cancer, colorectal cancer (CRC), prostate cancer, hepatocellular carcinoma (HCC), and head and neck squamous cell carcinoma (HNSCC) are the most common cancers in the world. However, the etiology underlying cancer development is far from comprehensively demonstrated^1^. Gene mutations, such as single nucleotide polymorphism (SNP), have been indicated in recent years to have an impact on the susceptibility of cancer^2,3^.

*Fibroblast growth factor receptor 4* (*FGFR4*) is a member of the family of fibroblast growth factor receptors. It displays a variety of biological activities, including angiogenic and mitogenic activities. It can also transduce signals of more than 20 known ligands, such as those involved in cell proliferation, differentiation and development^4,5^. *FGFR4* is highly activated in many types of solid tumors and hematological malignancies in which it drives the development and progression of cancer as an oncogene^6^. In addition, immunohistochemical evaluations have shown that the strong expression of *FGFR4* in malignant tumor cells is significantly correlated with an increase in clinical stage and tumor grade and a decrease in patients' survival rates^7^.

Recently, several studies have focused on the relationship between *FGFR4* gene variant and susceptibility of cancer^8–12^. A common SNP, rs351855, which leads to substitution of glycine by arginine at codon 388 in the domain of the FGFR4 receptor (Gly388Arg, G388R), has been reported to be associated with cancer risk. In 2017, a meta-analysis assessed *FGFR4* G388R polymorphism and found that it was correlated with an elevated susceptibility of several cancers^8^. Since then, more case–control studies have been conducted. Nevertheless, the correlation between *FGFR4* G388R, V10I variants, and susceptibility to carcinoma remains controversial. To comprehensively investigate the relationship between *FGFR4* G388R, V10I variants and cancer risk, we conducted the present analysis based on all eligible studies and used online databases and immunohistochemical staining (IHS) to assess the expression of FGFR4 further^9–33^.

## Methods

### Literature identification

A comprehensive search of eligible publications related to *FGFR4* G388R or V10I polymorphisms and cancer risk was performed using PubMed, EMbase, and the Chinese Wanfang database. The search terms were: (‘FGFR4’ OR ‘fibroblast growth factor receptor 4’) AND (‘variant’ OR ‘mutation’ OR ‘polymorphism’) AND (‘cancer’ OR ‘carcinoma’ OR ‘malignant tumor’). The latest search was conducted on May 2, 2020. The references of related reviews and original articles, as well as supplementary material, were also evaluated to maximize the coverage of the present analysis.

### Inclusion criteria and exclusion criteria

The inclusion criteria were as follows: (a) investigations of the relationship between *FGFR4* G388R or V10I polymorphisms and risk of cancer; (b) cohort or case–control studies; (c) sufficient genotype information to calculate Odds ratios (ORs) and 95% confidence intervals (95%CIs); and (d) *P*-values greater than 0.05 for Hardy–Weinberg equilibrium (HWE) of controls. Articles that departed from HWE were removed. We also excluded studies with no control population. When repeated studies appeared, only the latest or largest articles were included.

### Data extraction

Two authors independently searched the articles and extracted data from individual studies according to the inclusion criteria. Information collected from all eligible studies included the name of the first author, publication date, the ethnicity of subjects in the study, source of control, number of genotyped cases and controls, *P*-values for HWE of controls, and genotyping method. If a type of cancer appeared in only one study, then this cancer was classified in to ‘other cancer’ group. A total of 37 eligible studies were included.

### Statistical analysis

We adopted ORs with 95% CI to explore the correlation between *FGFR4* G388R or V10I polymorphisms and the risk of cancer. For the G388R variant, five genetic models were used (allelic contrast, R vs. G, heterozygous model, RG vs. GG, homozygous model, RR vs. GG; dominant model, RR + RG vs. GG, recessive model RR vs. RG + GG). For V10I polymorphism, the five models were as follows: I versus V; IV versus VV; II versus VV; II + IV versus VV; II versus IV + VV. The homogeneity of the study was calculated by a chi-square-based Q-test. *P*-value ≥ 0.05 indicated a lack of heterogeneity; the summary OR was evaluated by the fixed-effects model (Mantel–Haenszel method). Otherwise, the random-effects model was employed. Begg's funnel plot and sensitivity analysis were performed to assess publication bias. Stratification analysis was applied to evaluate the impact of ethnicity and cancer type. All statistical analyses were performed using Stata software (Stata Corporation 2009. *Stata Statistical Software: Release 11*. College Station, TX: StataCorp LP.).

### In silico and IHS analysis of FGFR4 expression

We employed an online database to assess the expression of *FGFR4* in prostate cancer and normal tissues (https://www.cancer.gov/about-nci/organization/ccg/research/structural-genomics/tcga). Moreover, gene–gene interaction of *FGFR4* was also evaluated by an online database (http://ualcan.path.uab.edu/analysis.html). The Cancer Genome Atlas (TCGA) samples were also applied to investigate the effect of *FGFR4* expression on overall survival (OS) time (http://genomics.jefferson.edu/proggene/results.php). The relationship between G388R or V10I polymorphisms and FGFR4 protein damage was analyzed by Polyphen2 tools (http://genetics.bwh.harvard.edu/pph2/).

Furthermore, we used IHS to test the tissue expression of FGFR4 in prostate cancer subjects recruited by our centers. From February 2013 to July 2018, a total of 220 patients diagnosed with prostate cancer by puncture biopsy were enrolled in our study. These patients underwent radical prostatectomy in our hospitals. Before the IHS analysis, each participant signed an informed consent. In addition to the routine pathological examination, the remaining part of the tissue removed during the operation was used for immunohistochemical examination. Paraffin-embedded samples were stained with hematoxylin and eosin to confirm cancer. Tissue sections were dewaxed in xylene, dehydrated in alcohol and washed in phosphate buffer (PBS). Each slice was incubated overnight with rabbit anti-FGFR4 monoclonal antibody at 4 °C. After being rinsed with PBS for three times, the slices were incubated with secondary antibody at 20 °C for 30 min. PBS was used instead of a primary antibody as a negative control. Two authors evaluated the prostate cancer sections separately. We investigated the intensities of FGFR4 reactivities in different samples utilizing the image J software (Version 1.45, a java-based image analysis program designed by National Institutes of Health, Bethesda, Maryland, USA, Available from: URL: https://rsb.info.nih.gov/ij/) (range from score 1 to 8)^34,35^. The present study was approved by Ethics Committee of Changzhou No.2 People's Hospital and Affiliated Hospital of Jiangnan University.

### Ethical approval and consent to participate

The present research was approved by Ethics Committee of Changzhou No.2 People's Hospital and Affiliated Hospital of Jiangnan University.

## Results

### Characteristics of included studies

**Figure 1:**
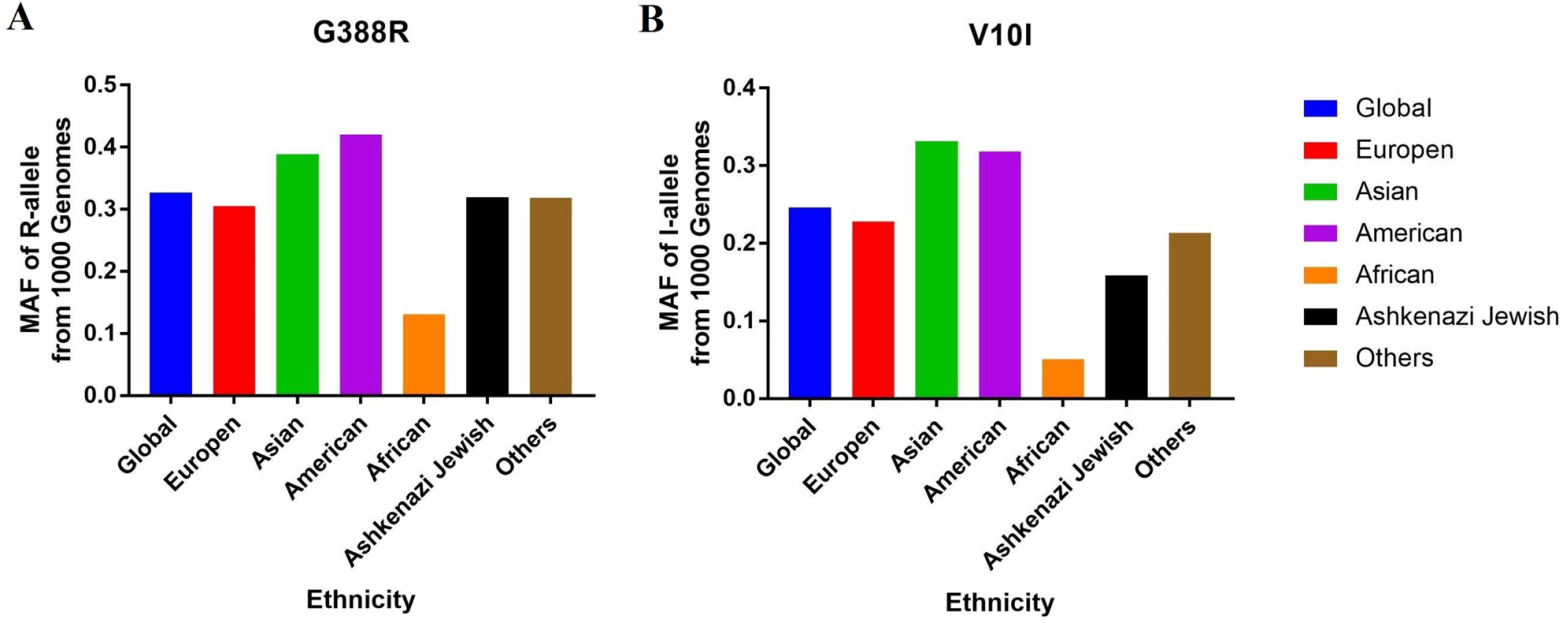
Minor allele frequencies of *FGFR4* G388R (**A**) and V10I (**B**) variants in various races.

A total of 269 articles were initially involved based on the inclusion criteria (Supplement Fig. 1). After screening the abstracts, we excluded 95 articles. Then, another 149 articles were removed because they were reviews, duplicates, or studies with no control group or focus on other SNPs. Finally, 25 eligible articles (with 37 studies) concerning *FGFR4* G388R or V10I polymorphisms were included in our pooled analysis. Data were collected on 29,972 participants (13,793 cancer subjects and 16,179 controls) from 30 case–control studies on *FGFR4* G388R polymorphism. The most common types of cancer were prostate cancer (6 studies, n = 4610), breast cancer (6 studies, n = 3008), hepatocellular carcinoma (HCC) (3 studies, n = 2481), oral squamous cell carcinoma (OSCC) (2 studies, n = 2396), colorectal cancer (CRC) (3 studies, n = 2349), lung cancer (2 studies, n = 899), cervical cancer (2 studies, n = 885) and other cancers (6 studies, n = 3975) (Table 1). Subgroup analysis by ethnicity evaluated 15 studies on European populations, 13 studies on Asians, one study on Latin Americans, and one study on Africans. For the *FGFR4* V10I variant, seven studies with 9369 subjects (4377 cases and 4992 controls) in total were identified, and of these, four studies focused on Asians (n = 5055), two on Europeans (n = 4087), and one on Africans (n = 227). In the stratification analysis by cancer type, two studies concentrated on prostate cancer. There was only one study each on cervical cancer, OSCC, breast cancer, HCC, and skin cancer. Furthermore, we checked the minor allele frequencies (MAF) of the worldwide population reported from gnomAD database. For *FGFR4* G388R polymorphism, the frequency in the global population was 0.327; African descendants, 0.131; Americans, 0.420; Asians, 0.389; Europeans, 0.294; Ashkenazi Jewish, 0.320; and others, 0.318 (Fig. 1A). For the *FGFR4* V10I variant, the frequency in the global population was 0.246; Africans, 0.051; Americans, 0.319; Asians, 0.332; Europeans, 0.228; Ashkenazi Jewish, 0.159; and others, 0.213 (Fig. 1B).

**Table 1:** Basic information of included studies for *FGFR4* G388R, V10I variants and risk of cancer.

First author G388R	Year	Origin	Cancer	Ethnicity	Source	Case	Control	Case			Control			HWE	Method
								RR	RG	GG	RR	RG	GG		
Wimmer	2019	Germany	HNSCC	European	PB	284	123	12	84	188	8	60	55	0.114	PCR–RFLP
Chen	2018	Taiwan	Cervical cancer	Asianfb	HB	226	335	56	101	69	74	165	96	0.845	TaqMan
Li	2017	China mainland	Cervical cancer	Asian	HB	162	162	48	79	35	40	72	50	0.170	PCR–RFLP
Chou	2017	Taiwan	OSCC	Asian	PB	955	1191	206	524	225	334	596	261	0.873	TaqMan
Sheu	2015	China mainland	HCC	Asian	HB	289	595	57	150	82	122	314	159	0.146	TaqMan
Jiang	2015	China mainland	Breast cancer	Asian	NA	747	716	138	404	205	98	348	270	0.398	Snapshot
Ture	2015	Turkey	Lung cancer	European	HB	124	100	11	47	66	6	46	48	0.242	PCR–RFLP
Gao	2014	China mainland	NHL	Asian	NA	421	486	115	189	117	75	240	171	0.541	PCR–RFLP
Shen	2013	China mainland	Gastric cancer	Asian	PB	304	392	62	124	118	72	188	132	0.724	Sequencing
Heinzle	2012	Austria	CRC	European	PB	85	1660	10	33	42	135	723	802	0.114	TaqMan
Yang	2012	China mainland	HCC	Asian	HB	711	740	144	351	216	132	361	247	0.996	TaqMan
Batschauer	2011	Brazil	Breast cancer	Latin	PB	68	85	3	26	39	3	35	47	0.249	PCR–RFLP
Ho	2010	UK	Prostate cancer	European	PB	397	291	32	182	183	24	117	150	0.860	TaqMan
Tanuma	2010	Japan	OSCC	Asian	HB	150	100	28	53	69	10	48	42	0.487	PCR-SSCP
FitzGerald	2009	USA	Prostate cancer	European	PB	1254	1251	123	544	587	124	496	631	0.070	SNPlex
FitzGerald	2009	USA	Prostate cancer	African	PB	146	80	3	39	104	2	18	60	0.646	SNPlex
Ho	2009	Singapore	HCC	Asian	PB	58	88	14	17	27	20	38	30	0.241	Sequencing
Naidu	2009	Malaysia	Breast cancer	Asian	HB	387	252	36	172	179	15	105	132	0.322	PCR–RFLP
Nan	2009	USA	Skin cancer	European	PB	768	833	78	325	365	84	343	406	0.359	TaqMan
Ma	2008	Japan	Prostate cancer	Asian	HB	492	179	133	196	163	25	87	67	0.701	PCR–RFLP
Mawrin	2006	Germany	Glioma	European	HB	94	25	4	51	39	2	13	10	0.428	PCR–RFLP
Spinola	2005	Italy	Lung cancer	European	HB	274	401	22	104	148	40	168	193	0.699	Pyrosequencing
Spinola	2005	Italy	Breast cancer	European	HB	142	220	20	55	67	25	83	112	0.117	Pyrosequencing
Spinola	2005	Italy	CRC	European	HB	179	220	18	63	98	25	83	112	0.117	Pyrosequencing
Wang	2004	USA	Prostate cancer	European	PB	284	97	42	117	125	4	40	53	0.291	PCR–RFLP
Wang	2004	USA	Prostate cancer	European	PB	45	94	2	6	37	0	18	76	0.305	PCR–RFLP
Morimoto	2003	Japan	Sarcomas	Asian	NA	143	102	17	72	54	13	50	39	0.624	PCR–RFLP
Bange	2002	Russia	Breast cancer	European	PB	61	123	7	28	26	8	60	55	0.114	PCR–RFLP
Bange	2002	Germany	Breast cancer	European	PB	84	123	9	34	41	8	60	55	0.114	PCR–RFLP
Bange	2002	Italy	CRC	European	PB	82	123	7	38	37	8	60	55	0.114	PCR–RFLP
V10I								II	IV	VV	II	IV	VV		
Chen	2018	Taiwan	Cervical cancer	Asian	HB	227	335	61	105	61	76	168	91	0.927	TaqMan
Chou	2017	Taiwan	OSCC	Asian	PB	955	1191	228	514	213	326	580	285	0.391	TaqMan
Jiang	2015	China mainland	Breast cancer	Asian	NA	747	716	168	408	171	226	364	126	0.322	Snapshot
Sheu	2015	China mainland	HCC	Asian	HB	289	595	64	160	65	144	300	151	0.835	TaqMan
Nan	2009	USA	Skin cancer	European	PB	753	821	41	251	461	43	271	507	0.390	TaqMan
FitzGerald	2009	USA	Prostate cancer	European	PB	1259	1254	72	405	782	65	447	742	0.827	SNPlex
FitzGerald	2009	USA	Prostate cancer	African	PB	147	80	0	15	132	0	10	70	0.551	SNPlex

CRC, colorectal cancer; HCC, hepatocellular carcinoma; HNSCC, head and neck squamous cell carcinoma; OSCC, oral squamous cell carcinoma; HB, hospital-based; HWE, Hardy–Weinberg equilibrium of controls; NHL, non-Hodgkin's lymphoma; NA, not available; PCR–RFLP, polymerase chain reaction and restrictive fragment length polymorphism; PB, population-based.

### Main results

**Figure 2:**
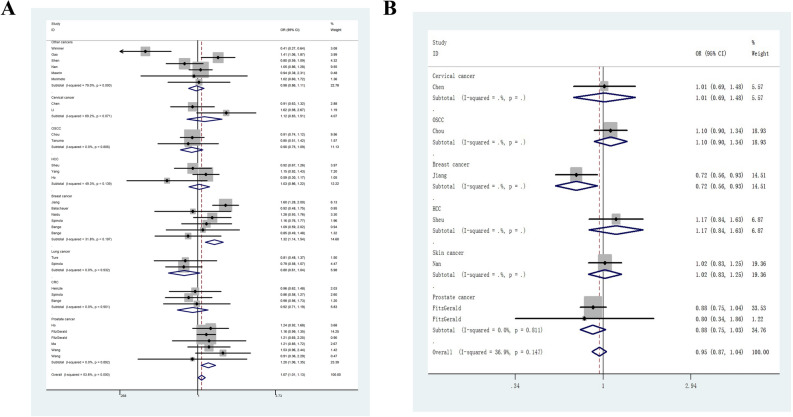
Forest plot of odds ratio for the association between *FGFR4* G388R (**A**) and V10I (**B**) polymorphisms and susceptibility of cancer (dominant genetic model, fixed-effects) in subgroup analysis by cancer type.

**Figure 3:**
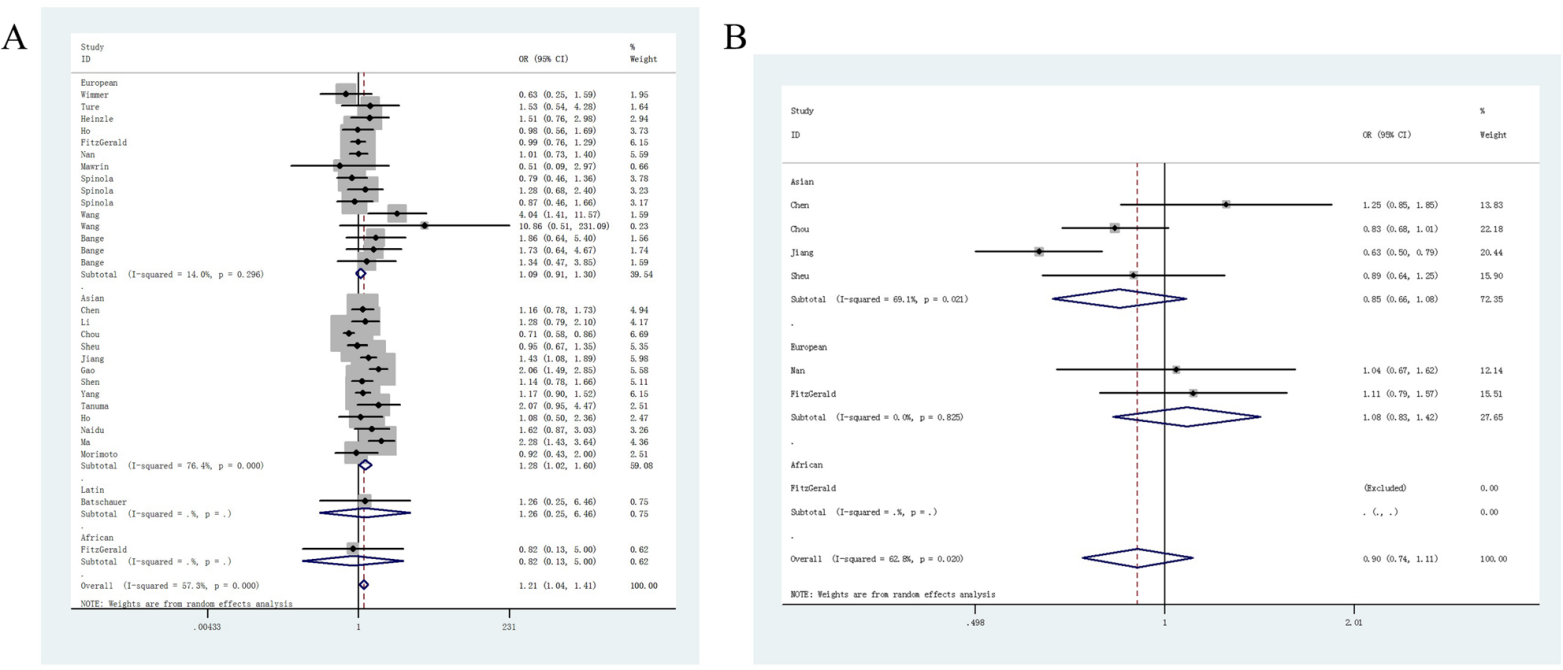
Stratified analysis by ethnicity between *FGFR4* G388R (**A**), V10I (**B**) polymorphisms and cancer risk (recessive genetic model, random-effects).

The overall results showed that the *FGFR4* G388R variant was associated with elevated susceptibility to cancer under homozygous comparison (OR = 1.21, 95%CI = 1.03–1.43, *P*_heterogeneity_ < 0.001, *P* = 0.020) and recessive genetic modeling (OR = 1.21, 95%CI = 1.04–1.41, *P* value for heterogeneity < 0.001, *P* = 0.012, Table 2). The stratification analysis by cancer type revealed that individuals with the RR + RG allele had a 1.20-fold higher susceptibility to prostate cancer than those with the GG allele (95%CI = 1.06–1. 35, *P*_heterogeneity_ = 0.892, *P* = 0.004, Fig. 2A). Individuals with the RR + RG allele had a 1.26-fold higher risk of breast cancer than those with the wild type (95%CI = 1.14–1.54, *P*_heterogeneity_ = 0.197, *P* < 0.001). In subgroup analysis by ethnicity, we observed that Asian descendants carrying the RR allele had a 1.28-fold increased risk of cancer compared with those carrying the RG + GG allele (95%CI = 1.02–1.60, *P*_heterogeneity_ < 0.001, *P* = 0.034, Fig. 3A). However, we did not identify positive results in European (95%CI = 0.93–1.26, *P* = 0.306), African (95%CI = 0.13–5.00, *P* = 0.828), or Latin Americans (95%CI = 0.25–6.46, *P* = 0.780). For *FGFR4* V10I polymorphism, no significant correlation was found when all studies were pooled (I vs. V, OR = 0.94, 95%CI = 0.85–1.04, *P* value for heterogeneity = 0.049, *P* = 0.227; IV vs. VV, OR = 0.97, 95%CI = 0.89–1.07, *P*_heterogeneity_ = 0.169, *P* = 0.601; II vs. VV, OR = 0.92, 95%CI = 0.72–1.17, *P* value for heterogeneity = 0.020, *P* = 0.488; II + IV vs. VV, OR = 0.95, 95%CI = 0.87–1.04, *P* value for heterogeneity = 0.147, *P* = 0.300, Fig. 2B; II vs. IV + VV, OR = 0.90, 95%CI = 0.74–1.11, *P* value for heterogeneity = 0.020, *P* = 0.328). Similar findings were indicated in the subgroup analysis by cancer type. In stratification analysis by ethnicity, we observed that *FGFR4* V10I mutation may not have an impact on the risk of cancer for Asian (95%CI = 0.66–1.08, *P* = 0.184, Fig. 3B), African (95%CI = 0.34–1.86, *P* = 0.598), or individuals with European descent (95%CI = 0.83–1.42, *P* = 0.563).

**Table 2:** Stratified analysis of *FGFR4* G388R or V10I polymorphisms on susceptibility of cancer.

Variables	N^a^	Case/control	M-allele versus W-allele			MW versus WW			MM versus WW			MM + MW versus WW			MM versus MW + WW		
			OR (95%CI)	*P*_heter_	*P*	OR (95%CI)	*P*_heter_	*P*	OR (95%CI)	*P*_heter_	*P*	OR (95%CI)	*P*_heter_	*P*	OR (95%CI)	*P*_heter_	*P*
**G388R**																	
Total	30	9416/11187	1.06 (0.98–1.15)	< 0.001	0.123	0.99 (0.90–1.09)	0.003	0.820	1.21 (1.03–1.43)	< 0.001	0.020	1.03 (0.94–1.14)	< 0.001	0.526	1.21 (1.04–1.41)	< 0.001	0.012
**Ethnicity**																	
Asian	13	5045/5338	1.12 (0.99–1.26)	< 0.001	0.078	1.03 (0.89–1.18)	0.011	0.715	1.29 (1.01–1.66)	< 0.001	0.043	1.09 (0.94–1.26)	0.002	0.247	1.28 (1.02–1.60)	< 0.001	0.034
European	15	4157/5684	1.01 (0.90–1.13)	0.013	0.876	0.94 (0.81–1.09)	0.016	0.396	1.10 (0.94–1.28)	0.198	0.238	0.97 (0.84–1.12)	0.010	0.669	1.08 (0.93–1.26)	0.296	0.306
African	1	146/80	1.14 (0.66–1.98)	–	0.635	1.25 (0.66–2.38)	–	0.496	0.87 (0.14–5.33)	–	0.876	1.21 (0.65–2.25)	–	0.544	0.82 (0.13–5.00)	–	0.828
LA	1	68/85	0.97 (0.57–1.64)	–	0.905	0.90 (0.46–1.73)	–	0.743	1.21 (0.23–6.31)	–	0.825	0.92 (0.48–1.75)	–	0.799	1.26(0.25–6.46)	–	0.780
**Cancer type**																	
Prostate cancer	6	2618/1992	1.17 (1.07–1.29)	0.183	0.001	1.16 (1.02–1.32)	0.714	0.025	1.60 (0.98–2.61)	0.020	0.058	1.20 (1.06–1.35)	0.892	0.004	1.56 (0.92–2.65)	0.004	0.103
Cervical cancer	2	388/497	1.12 (0.93–1.36)	0.178	0.225	1.06 (0.77–1.45)	0.073	0.729	1.26 (0.88–1.82)	0.209	0.211	1.12 (0.83–1.51)	0.071	0.454	1.21 (0.89–1.65)	0.756	0.228
OSCC	2	1105/1291	0.87 (0.78–0.98)	0.188	0.019	0.97 (0.79–1.18)	0.164	0.726	1.01 (0.44–2.32)	0.046	0.984	0.90 (0.75–1.09)	0.806	0.284	1.13 (0.40–3.21)	0.008	0.820
HCC	3	1058/1423	1.04 (0.93–1.16)	0.241	0.518	1.00 (0.83–1.20)	0.127	0.991	1.09 (0.86–1.37)	0.341	0.478	1.03 (0.86–1.22)	0.139	0.769	1.09 (0.89–1.33)	0.656	0.423
Breast cancer	6	1489/1519	1.26 (1.13–1.41)	0.622	< 0.001	1.25 (1.07–1.47)	0.186	0.005	1.73 (1.35–2.20)	0.960	< 0.001	1.32 (1.14–1.54)	0.197	< 0.001	1.46 (1.17–1.83)	0.986	0.001
Lung cancer	2	398/501	0.85 (0.69–1.05)	0.587	0.138	0.79 (0.60–1.05)	0.800	0.099	0.82 (0.50–1.34)	0.312	0.435	0.80 (0.61–1.04)	0.932	0.091	0.91 (0.57–1.47)	0.267	0.704
CRC	3	346/2003	0.98 (0.80–1.19)	0.691	0.820	0.89 (0.67–1.17)	0.972	0.387	1.08 (0.69–1.69)	0.521	0.730	0.9 2 (0.71–1.19)	0.901	0.518	1.14 (0.74–1.76)	0.496	0.551
Other cancers	6	2014/1961	0.96 (0.74–1.24)	< 0.001	0.743	0.85 (0.63–1.15)	0.003	0.301	1.04 (0.67–1.63)	0.003	0.854	0.89 (0.65–1.23)	< 0.001	0.496	1.13 (0.78–1.64)	0.012	0.510
**V10I**																	
Total	7	4377/4992	0.94 (0.85–1.04)	0.049	0.227	0.97 (0.89–1.07)	0.169	0.601	0.92 (0.72–1.17)	0.020	0.488	0.95 (0.87–1.04)	0.147	0.300	0.90 (0.74–1.11)	0.020	0.328
**Ethnicity**																	
Asian	4	2218/2837	0.93 (0.79–1.10)	0.011	0.408	1.05 (0.91–1.21)	0.140	0.493	0.87 (0.62–1.22)	0.009	0.423	0.98 (0.78–1.23)	0.049	0.844	0.85 (0.66–1.08)	0.021	0.184
European	2	2012/2075	0.97 (0.87–1.07)	0.407	0.516	0.92 (0.80–1.05)	0.220	0.200	1.05 (0.80–1.38)	0.994	0.728	0.93 (0.82–1.06)	0.269	0.293	1.08 (0.83–1.42)	0.825	0.563
African	1	147/80	0.81 (0.35–1.84)	–	0.609	0.80 (0.34–1.86)	–	0.598	NA			0.80 (0.34–1.86)	–	0.598	NA		
**Cancer type**																	
Prostate cancer	2	1406/1334	0.93 (0.82–1.06)	0.732	0.276	0.86 (0.73–1.01)	0.861	0.067	1.05 (0.74–1.49)	–	0.780	0.88 (0.75–1.03)	0.811	0.114	1.11 (0.79–1.57)	–	0.555
Cervical cancer	1	227/335	1.09 (0.86–1.39)	–	0.461	0.93 (0.62–1.40)	–	0.735	1.20 (0.75–1.91)	–	0.450	1.01 (0.69–1.48)	–	0.939	1.25 (0.85–1.85)	–	0.257
OSCC	1	955/1191	0.96 (0.85–1.09)	–	0.542	1.19 (0.96–1.47)	–	0.118	0.94 (0.73–1.20)	–	0.596	1.10 (0.90–1.34)	–	0.375	0.83 (0.68–1.01)	–	0.066
Breast cancer	1	747/716	0.75 (0.65–0.87)	–	< 0.001	0.83 (0.63–1.08)	–	0.165	0.55 (0.40–0.74)	–	< 0.001	0.72 (0.56–0.93)	–	0.012	0.63 (0.50–0.79)	–	< 0.001
HCC	1	289/595	1.02 (0.83–1.24)	–	0.870	1.24 (0.87–1.76)	–	0.228	1.03 (0.68–1.55)	–	0.880	1.17 (0.84–1.63)	–	0.349	0.89 (0.64–1.25)	–	0.499
Skin cancer	1	753/821	1.02 (0.86–1.21)	–	0.802	1.02 (0.82–1.26)	–	0.865	1.05 (0.67–1.64)	–	0.835	1.02 (0.83–1.25)	–	0.828	1.04 (0.67–1.62)	–	0.855

CRC, colorectal cancer; HCC, hepatocellular carcinoma; HNSCC, head and neck squamous cell carcinoma; LA, Latin Americans; NA, not available; OSCC: oral squamous cell carcinoma.

*P*_heter_: *P* value of *Q*-test for heterogeneity test.

^a^ Number of comparisons.

### In silico and IHS analyses of FGFR4 expression

**Figure 4:**
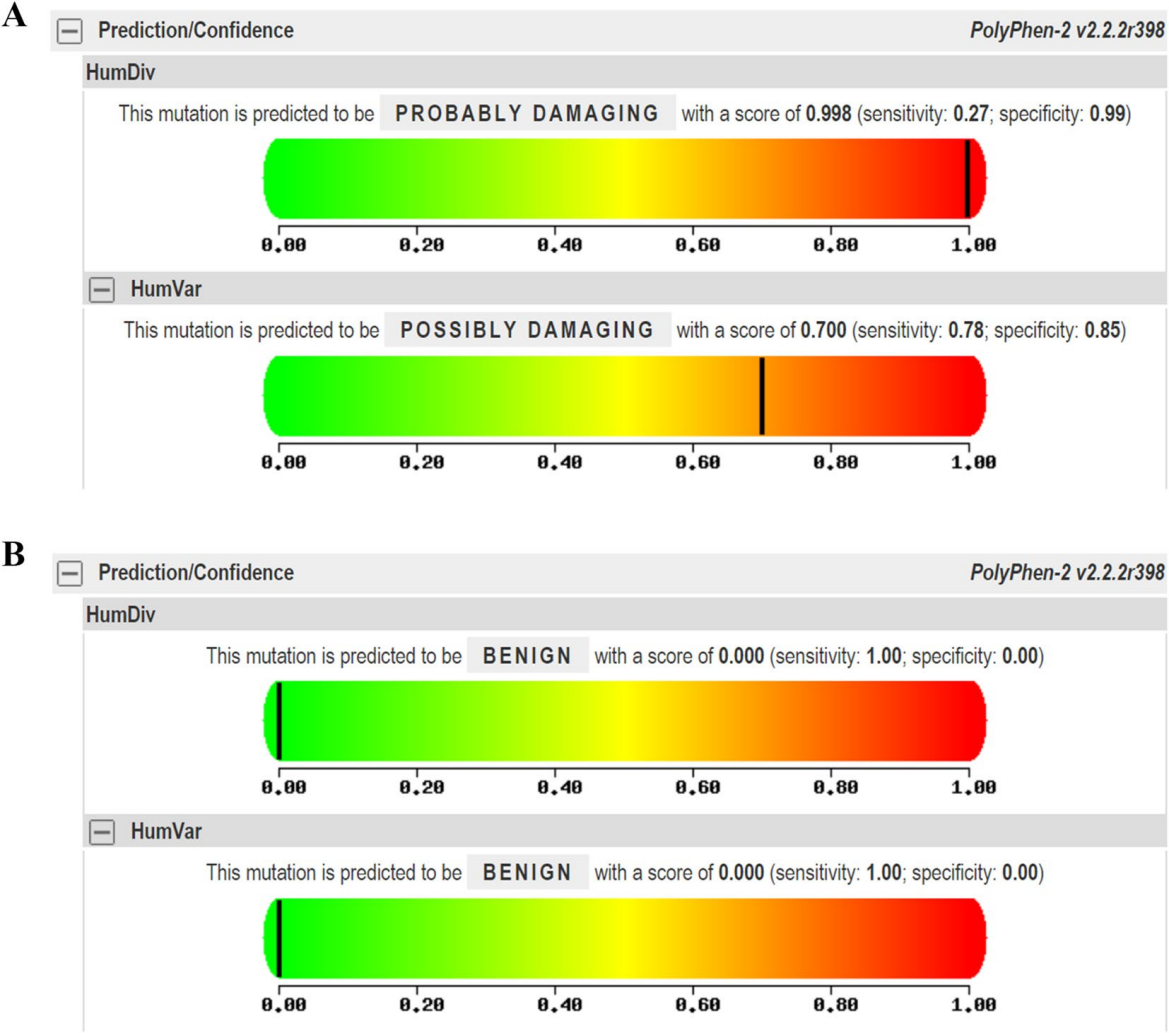
The relationship between G388R, V10I polymorphisms and FGFR4 protein damaging analyzed by Polyphen2 tools (black lines represent scores).

**Figure 5:**
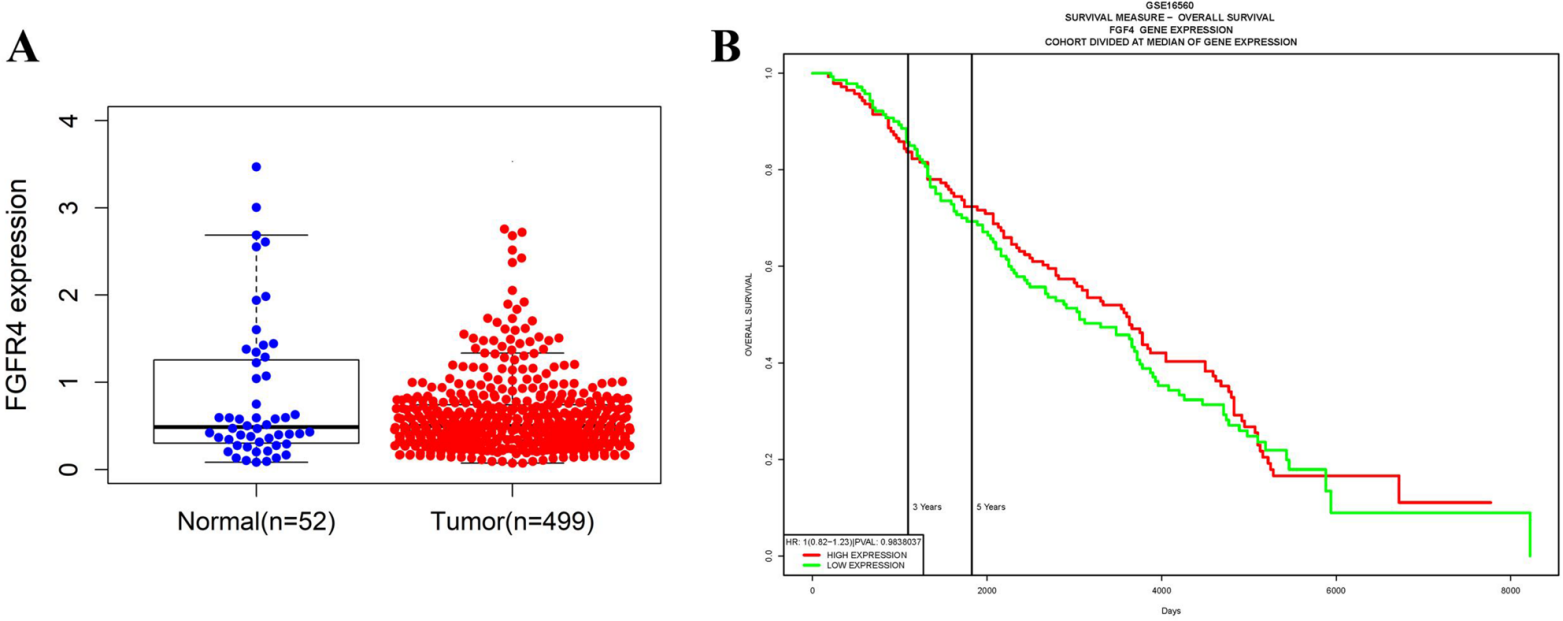
In silico analysis of *FGFR4* expression in prostate cancer patients (**A**). Effect of *FGFR4* expression level on prostate cancer participant’s overall survival (OS) time (**B**).

We used in silico tools to investigate whether G388R and V10I mutations affect the protein function of FGFR4. Polyphen2 bioinformatics analysis showed that *FGFR4* G388R was predicted to damage protein function, with a score of 0.700 (Fig. 4A). However, the V10I variation was predicted to be benign, with a score less than 0.001 (Fig. 4B). We also utilized an online database to assess the expression of *FGFR4* in prostate cancer participants and normal controls. As described in Fig. 5A, *FGFR4* expression is elevated in prostate cancer compared with that in the control. TCGA samples were also analyzed to investigate the effect of *FGFR4* expression on OS time. No significant difference in OS time was observed between the high *FGFR4* expression group and the low expression group (*P* > 0.05, Fig. 5B).

**Figure 6:**
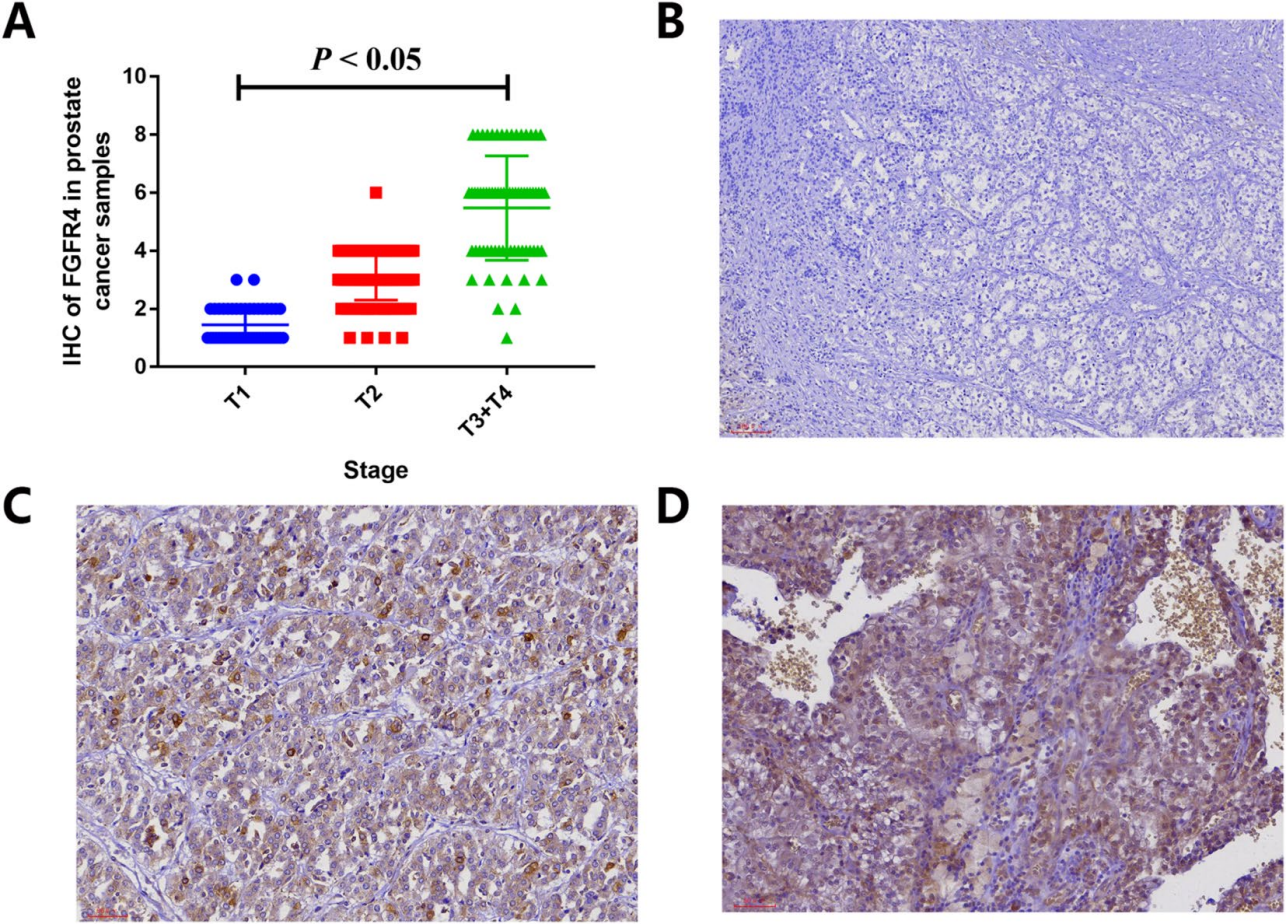
Tissue expression of FGFR4 among prostate cancer participants. The intensity of immunoreactivity was mainly concentrated in the cytoplasm of prostate cancer epithelial cells (**C**,**D**). The expression of FGFR4 is up-regulated in more advanced cases (**D**), as compared to ones in early stage (**A**,**B**, *P* < 0.05).

**Figure 7:**
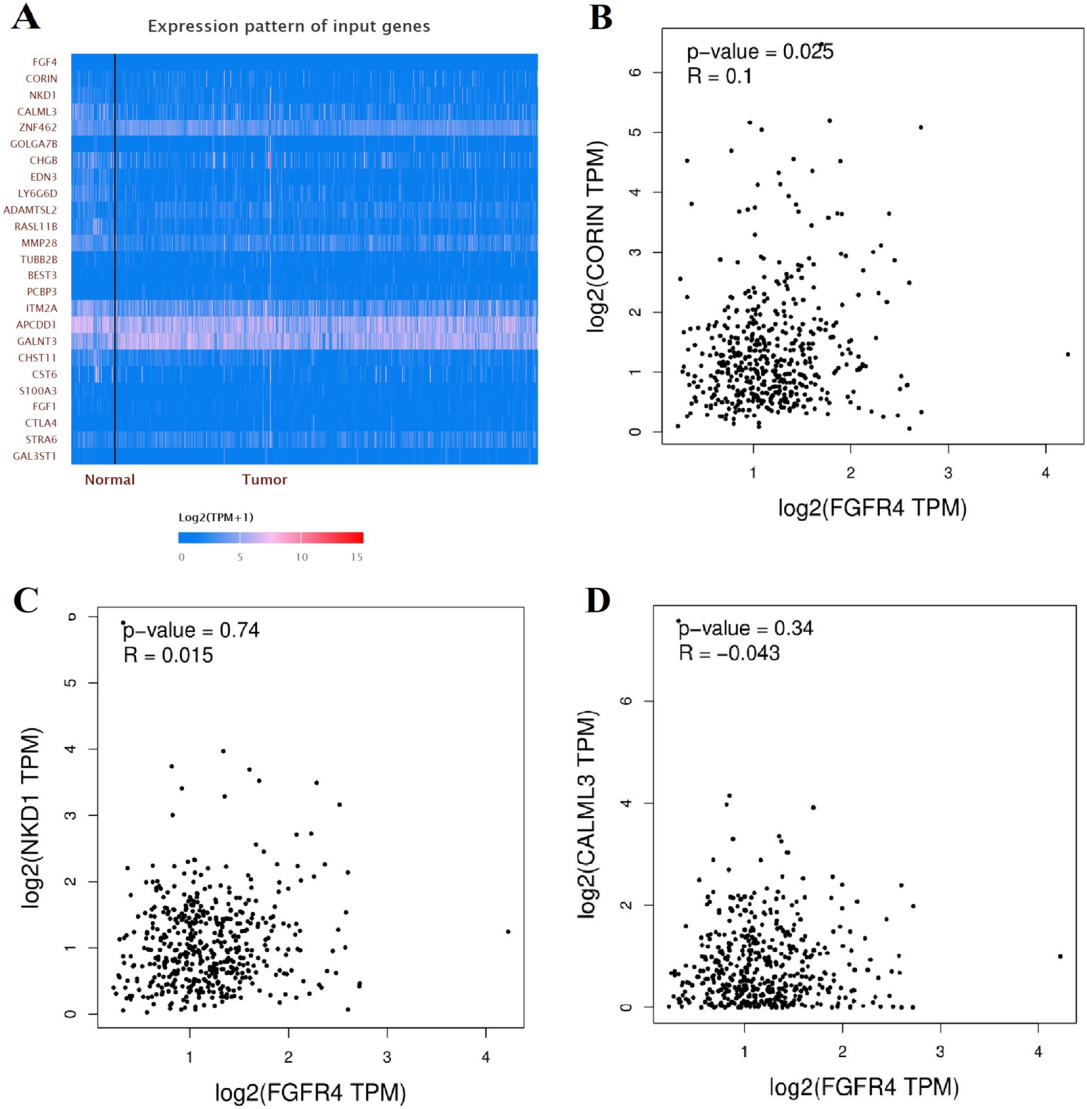
Gene–gene interaction of *FGFR4* in prostate cancer (**A**). Correlation analysis by TCGA samples show that *CORIN* (corin, serine peptidase, **B**) is predicted to positively correlated with *FGFR4*. The interaction between *NKD1* (Naked1, NKD inhibitor of *WNT* signaling pathway 1) and *FGFR4* is shown in (**C**). The correlation between *CALML3* (calmodulin like 3) and *FGFR4* is described in (**D**).

In order to demonstrate the expression of *FGFR4* in prostate cancer tissues, we applied IHS to evaluate its expression among prostate cancer patients at our centers. A total of 220 prostate cancer participants were enrolled in our centers. The feature distribution from prostate cancer volunteers has been provided in our previous article^36^. Immunohistochemistry of FGFR4 in prostate cancer specimens is described in Fig. 6. The intensity of immunoreactivity was mainly concentrated in the cytoplasm of prostate cancer epithelial cells (Fig. 6C,D). The expression of *FGFR4* was up-regulated in more advanced cases (Fig. 6D) compared with early stage cases (Fig. 6A,B, *P* < 0.05). Moreover, the gene–gene correlation of *FGFR4* was also assessed. At least 24 genes were shown to participate in interactions with *FGFR4* (Fig. 7A). The most related genes to *FGFR4* include: *CORIN* (corin, serine peptidase, Fig. 7B), *NKD1* (Naked1, NKD inhibitor 1, Fig. 7C), and *CALML3* (calmodulin like 3, Fig. 7D).

### Publication bias and sensitivity analysis

**Figure 8:**
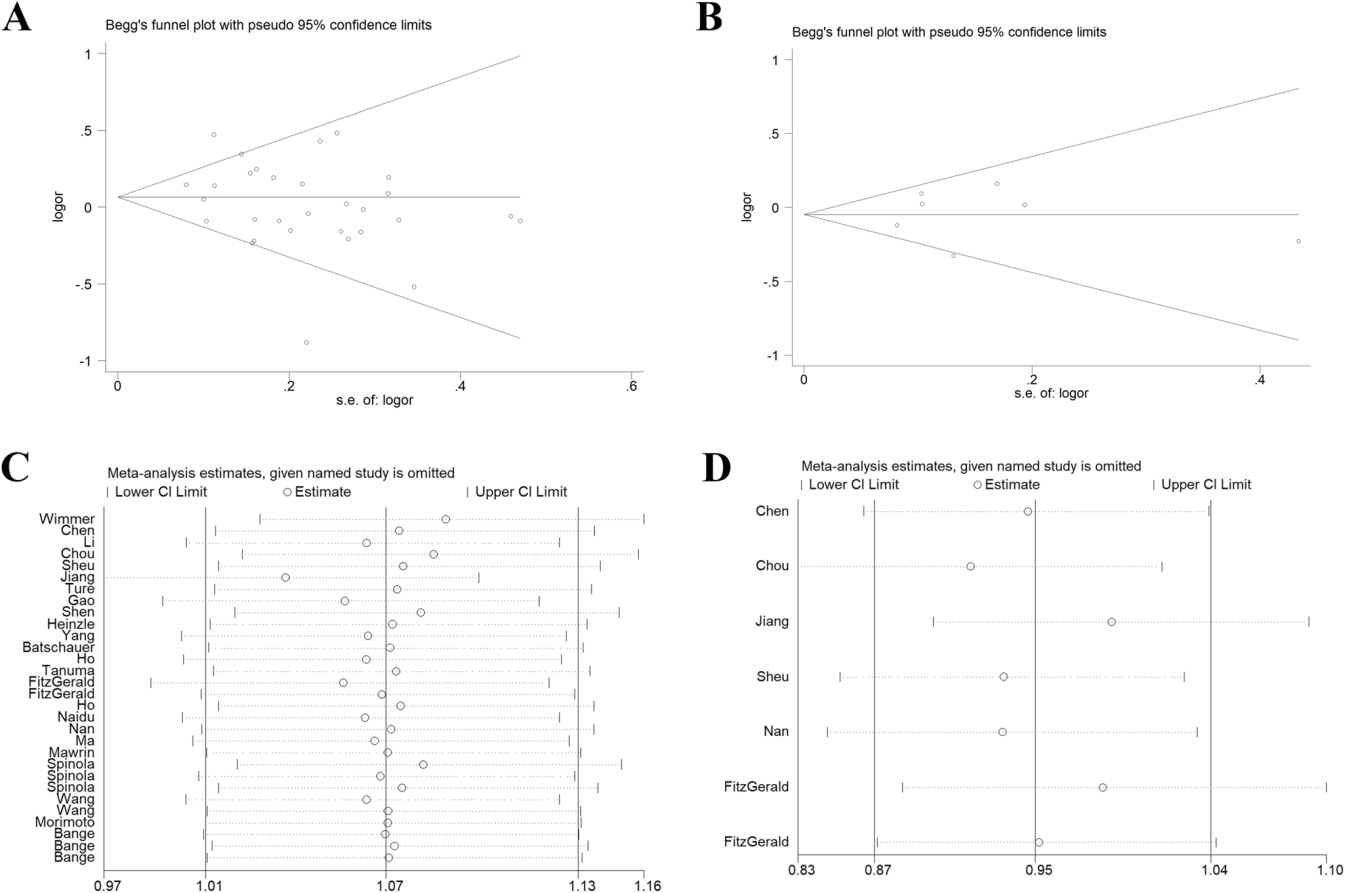
Begg's funnel plot analysis for *FGFR4* G388R (**A**) and V10I (**B**) polymorphisms under dominant genetic model. Sensitivity analysis for *FGFR4* G388R (**C**) and V10I (**D**) polymorphisms and risk of cancer. No evidence of publication bias was identified.

A Begg's funnel plot was employed to investigate publication bias. No evidence of asymmetry was observed for *FGFR4* G388R (t =  − 1.52, *P* = 0.140, Fig. 8A) or V10I variants (t = 0.07, *P* = 0.945, Fig. 8B). Sensitivity analysis of *FGFR4* G388R or V10I polymorphisms and the risk of cancer was performed by removing individual studies in turn. No single study influenced the overall OR, indicating that the results of the above analysis for *FGFR4* G388R (Fig. 8C) and V10I (Fig. 8D) polymorphisms are reliable.

## Discussion

The etiology of cancer has not been totally elucidated. Clinically, SNPs have various influences on the development of diseases, including cancer^37–39^. The association between *FGFR4* G388R or V10I variants and the susceptibility of cancer has been evaluated previously, but the results are conflicting. For example, Ma et al. performed a case–control study and found that the R-allele of the G388R variant had a significant impact on the development and progression of prostate cancer in Japanese patients^27^. However, this conclusion could not be confirmed by the research of FitzGerald et al., who observed no positive correlation between *FGFR4* G388R or V10I polymorphisms and prostate cancer susceptibility in Caucasians or African Americans^23^. Xiong et al. performed a meta-analysis using articles published before October 2016 to assess the effect of the *FGFR4* G388R variant^8^. They showed that the G388R variant was correlated with increased susceptibility to prostate and breast cancer and reduced risk of lung cancer. In our analysis, all eligible studies based on inclusion criteria were included to extensively evaluate the association between *FGFR4* G388R or V10I variants and the susceptibility of cancer. We further adopted in silico and IHS analysis to confirm the above conclusion.

We performed a pooled analysis of studies that included 9416 cancer participants and 11,187 control subjects to investigate the relationship between the *FGFR4* G388R variant and susceptibility to cancer. In the current analysis, we found that G388R polymorphism is associated with an elevated risk of cancer. Furthermore, stratifying by type of cancer, we observed that this variant is correlated with prostate and breast cancer, but not with lung cancer. Our results are consistent with those of Wei et al. and Xu et al.^37,40^. In subgroup analysis by ethnicity, we also found that the *FGFR4* R-allele is correlated with an increased risk of cancer in individuals with Asian descent. For *FGFR4* V10I polymorphism, no significant relationship was indicated in either overall or stratifying analysis. The conclusions derived from our analysis were consistent with a previous meta-analysis published in 2010^41^. In 2017, another meta-analysis found that the *FGFR4* 388R variation was a reduced risk factor for lung cancer^8^. However, we did not come to this conclusion in the current analysis. The reason may be that there were few studies in our analysis that were focused on lung cancer^15,33^. Therefore, further well-designed studies with large sample sizes are needed to confirm the role of *FGFR4* G388R polymorphisms in lung cancer in future. Furthermore, we employed an in silico tool to investigate whether the G388R and V10I mutations could affect the protein function of *FGFR4*. It showed that the G388R mutation, but not the V10I mutation, could damage the protein function of FGFR4. We further utilized TCGA samples to assess the expression of FGFR4 in prostate cancer participants. The FGFR4 expression was elevated in prostate cancer compared with that in the control group. Nevertheless, no significant difference in the OS time could be identified between the high FGFR4 expression group and the low expression group. In addition, we applied IHS to evaluate its expression among prostate cancer subjects in our centers. The expression of FGFR4 is increased in more advanced cases, which indicates that up-regulation of FGFR4 is related to a poor prognosis of prostate cancer.

There are some potential limitations in the present analysis. First, the *P*-value of heterogeneity was less than 0.05 in five genetic models when all studies were pooled to assess *FGFR4* G388R polymorphism. Although the Der Simonian and Laird method (random-effect model) was used^42^, the analysis may have been influenced by potential bias. Second, the number of eligible studies on the *FGFR4* V10I variant in the present analysis remains insufficient for a comprehensive analysis. In our subgroup analysis by cancer type, only two studies concentrated on prostate cancer. A very limited number of studies were available for multiple types of cancers such as cervical cancer, OSCC, breast cancer, HCC, and skin cancer. Further research including more participants with various carcinomas is warranted to confirm this effect. Third, a previous study demonstrated that *FGFR4* G388R polymorphism was related to up-regulation of *FGFR4* in breast cancer^41^. For prostate cancer, further in vitro experiments are required to confirm the results from our pooled analysis. More functional research is warranted to determine whether the G388R mutation is responsible for increased expression of *FGFR4*. Moreover, genotyping the same patients will provide more persuasive evidence of a correlation between genotype or alleles and tissue expression of *FGFR4* observed by IHS analysis. Finally, at least 24 genes are involved in the interaction with *FGFR4*. Since few studies on these specific relationships can be retrieved from an online database, future studies are needed to ascertain these correlations in more detail.

In summary, our study showed that *FGFR4* G388R polymorphism is associated with an elevated risk of cancer, especially for prostate and breast cancer. The R-allele of the *FGFR4* G388R variant is correlated with an increased risk of cancer in individuals with Asian descent. The G388R mutation, but not the V10I mutation, is predicted to damage the protein function of FGFR4. Up-regulation of FGFR4 may be related to a poor prognosis in prostate cancer. These findings may guide personalized treatment of certain types of cancers.

## Supplementary Information

Supplementary Figure.

## Data Availability

All the data generated in the above research can be acquired from the corresponding authors upon reasonable request. All methods were conducted in accordance with relevant guidelines and regulations.

## Abbreviations

HNSCC: Head and neck squamous cell carcinoma
HCC: Hepatocellular carcinoma
OSCC: Oral squamous cell carcinoma
CRC: Colorectal cancer
HWE: Hardy–Weinberg equilibrium of controls
HB: Hospital-based
LA: Latin Americans
NA: Not available
NHL: Non-Hodgkin's lymphoma
PB: Population-based
PCR–RFLP: Polymerase chain reaction and restrictive fragment length polymorphism

## References

[1] Torre LA (2015). Global cancer statistics, 2012. CA Cancer J. Clin..

[2] Shao HB (2018). Human methionine synthase A2756G polymorphism increases susceptibility to prostate cancer. Aging (Albany NY).

[3] Zhang LF (2019). VEGF gene rs3025039C/T and rs833052C/A variants are associated with bladder cancer risk in Asian descendants. J. Cell Biochem..

[4] Wilkie AO, Morriss-Kay GM, Jones EY, Heath JK (1995). Functions of fibroblast growth factors and their receptors. Curr. Biol..

[5] Powers CJ, McLeskey SW, Wellstein A (2000). Fibroblast growth factors, their receptors and signaling. Endocr. Relat. Cancer.

[6] Tang S, Hao Y, Yuan Y, Liu R, Chen Q (2018). Role of fibroblast growth factor receptor 4 in cancer. Cancer Sci..

[7] Gowardhan B (2005). Evaluation of the fibroblast growth factor system as a potential target for therapy in human prostate cancer. Br. J. Cancer.

[8] Xiong SW (2017). Functional FGFR4 Gly388Arg polymorphism contributes to cancer susceptibility: evidence from meta-analysis. Oncotarget.

[9] Wimmer E (2019). Fibroblast growth factor receptor 4 single nucleotide polymorphism Gly388Arg in head and neck carcinomas. World J. Clin. Oncol..

[10] Chen TH (2018). Association of fibroblast growth factor receptor 4 genetic polymorphisms with the development of uterine cervical cancer and patient prognosis. Reprod. Sci..

[11] Li YP, Zhang L, Zou YL, Yu Y (2017). Association between FGFR4 gene polymorphism and high-risk HPV infection cervical cancer. Asian Pac. J. Trop. Med..

[12] Chou CH (2017). Functional FGFR4 Gly388Arg polymorphism contributes to oral squamous cell carcinoma susceptibility. Oncotarget.

[13] Sheu MJ (2015). Fibroblast growth factor receptor 4 polymorphism is associated with liver cirrhosis in hepatocarcinoma. PLoS ONE.

[14] Jiang Y (2015). Association of FGFR3 and FGFR4 gene polymorphisms with breast cancer in Chinese women of Heilongjiang province. Oncotarget.

[15] Ture M (2015). Investigation of FGFR4 (Gly388Arg) gene polymorphism in primary lung cancer patients. Int. J. Hum. Genet..

[16] Gao L (2014). Fibroblast growth factor receptor 4 polymorphism is associated with increased risk and poor prognosis of non-Hodgkin's lymphoma. Tumour. Biol..

[17] Shen YY (2013). Fibroblast growth factor receptor 4 Gly388Arg polymorphism in Chinese gastric cancer patients. World J. Gastroenterol..

[18] Heinzle C (2012). Differential effects of polymorphic alleles of FGF receptor 4 on colon cancer growth and metastasis. Cancer Res..

[19] Yang Y (2011). Association between fibroblast growth factor receptor 4 polymorphisms and risk of hepatocellular carcinoma. Mol. Carcinog..

[20] Batschauer AP (2011). HFE, MTHFR, and FGFR4 genes polymorphisms and breast cancer in Brazilian women. Mol. Cell Biochem..

[21] Ho CK, Anwar S, Nanda J, Habib FK (2010). FGFR4 Gly388Arg polymorphism and prostate cancer risk in Scottish men. Prostate Cancer Prostatic Dis..

[22] Tanuma J (2010). FGFR4 polymorphism, TP53 mutation, and their combinations are prognostic factors for oral squamous cell carcinoma. Oncol. Rep..

[23] FitzGerald LM (2009). Association of FGFR4 genetic polymorphisms with prostate cancer risk and prognosis. Prostate Cancer Prostatic Dis..

[24] Ho HK (2009). Fibroblast growth factor receptor 4 regulates proliferation, anti-apoptosis and alpha-fetoprotein secretion during hepatocellular carcinoma progression and represents a potential target for therapeutic intervention. J. Hepatol..

[25] Naidu R, Har YC, Taib NA (2009). Polymorphism of FGFR4 Gly388Arg does not confer an increased risk to breast cancer development. Oncol. Res..

[26] Nan H, Qureshi AA, Hunter DJ, Han J (2009). Genetic variants in FGFR2 and FGFR4 genes and skin cancer risk in the Nurses' Health Study. BMC Cancer.

[27] Ma Z (2008). Polymorphisms of fibroblast growth factor receptor 4 have association with the development of prostate cancer and benign prostatic hyperplasia and the progression of prostate cancer in a Japanese population. Int. J. Cancer.

[28] Mawrin C (2006). Analysis of a single nucleotide polymorphism in codon 388 of the FGFR4 gene in malignant gliomas. Cancer Lett..

[29] Spinola M (2005). FGFR4 Gly388Arg polymorphism and prognosis of breast and colorectal cancer. Oncol. Rep..

[30] Wang J, Stockton DW, Ittmann M (2004). The fibroblast growth factor receptor-4 Arg388 allele is associated with prostate cancer initiation and progression. Clin. Cancer Res..

[31] Morimoto Y (2003). Single nucleotide polymorphism in fibroblast growth factor receptor 4 at codon 388 is associated with prognosis in high-grade soft tissue sarcoma. Cancer.

[32] Bange J (2002). Cancer progression and tumor cell motility are associated with the FGFR4 Arg(388) allele. Cancer Res..

[33] Spinola M (2005). Functional FGFR4 Gly388Arg polymorphism predicts prognosis in lung adenocarcinoma patients. J. Clin. Oncol..

[34] Wirawati V (2019). The distribution of serotonergic nerve on the hippocampus of the fruit bats (*Rousettus amplexicaudatus*). Vet. World.

[35] Stanchev S (2020). Differential collagen expression in kidney and heart during hypertension. Bratisl. Lek. Listy..

[36] Zhang LF (2020). Association between SOD2 V16A variant and urological cancer risk. Aging (Albany NY).

[37] Wei W (2018). Prognostic implications of fibroblast growth factor receptor 4 polymorphisms in primary breast cancer. Mol. Carcinog..

[38] Dai F (2019). The association between three AXIN2 variants and cancer risk. J. Cell Biochem..

[39] Zhu L (2019). CDKN1B Val 109 Gly variant is not related to risk of prostate cancer. J. Cell Biochem..

[40] Xu B (2011). FGFR4 Gly388Arg polymorphism contributes to prostate cancer development and progression: a meta-analysis of 2618 cases and 2305 controls. BMC Cancer.

[41] Xu W (2010). FGFR4 transmembrane domain polymorphism and cancer risk: a meta-analysis including 8555 subjects. Eur. J. Cancer.

[42] DerSimonian R, Laird N (1986). Meta-analysis in clinical trials. Control Clin. Trials.

