# Supplementary material for: Effects of FGFR4 G388R, V10I polymorphisms on the likelihood of cancer
Source: Sci Rep. 2021 Jan 14;11:1373. doi: 10.1038/s41598-020-80146-y (PMC7809464; doi:10.1038/s41598-020-80146-y)

**Effects of *FGFR4* G388R, V10I polymorphisms on the likelihood of cancer**

Tao Peng^1*^, Yangyang Sun^2*^, Zhiwei Lv^3*^, Ze Zhang ^3*^, Quanxin Su^3*^, Hao Wu ^3^, Wei Zhang ^4^, Wei Yuan ^5^, Li Zuo ^3^, Li Shi ^3^, Li-Feng Zhang ^3^, Xiaoli Zhou^2^, Yuanyuan Mi ^1^

*^1^ Department of Urology, Affiliated Hospital of Jiangnan University, Wuxi 214000, People's Republic of China.*

^2^ *Department of Pathology, The Affiliated Changzhou No.2 People's Hospital of Nanjing Medical University, 29 Xinglong Road, Changzhou 213003, People's Republic of China.*

*^3^ Department of Urology, The Affiliated Changzhou No.2 People's Hospital of Nanjing Medical University, 29 Xinglong Road, Changzhou 213003, People's Republic of China.*

^4^ *Department of Oncology, Taizhou People's Hospital, South Hailing Road 399, Taizhou 225300, People's Republic of China.*

^5^ *Department of Cardiology, Taizhou People's Hospital, South Hailing Road 399, Taizhou 225300, People's Republic of China.*

*^*^Equal contributors.*

**Address correspondence to:** Li-Feng Zhang, Department of Urology, the Affiliated Changzhou No. 2 People's Hospital of Nanjing Medical University, 29 Xinglong Road, Changzhou 213003, People's Republic of China. E-mail: nj-likky@163.com; Xiaoli Zhou, Department of Pathology, The Affiliated Changzhou No.2 People's Hospital of Nanjing Medical University, 29 Xinglong Road, Changzhou 213003, People's Republic of China. E-mail: [tzuvmd@sohu.com;](mailto:tzuvmd@sohu.com;) Yuanyuan Mi, Department of Urology, Affiliated Hospital of Jiangnan University, Wuxi 214000, People's Republic of China. E-mail: miniao1984@163.com.

**Supplementary Figure 1: Flowchart of included studies on the association between *FGFR4* G388R or V10I polymorphisms on cancer risk.**


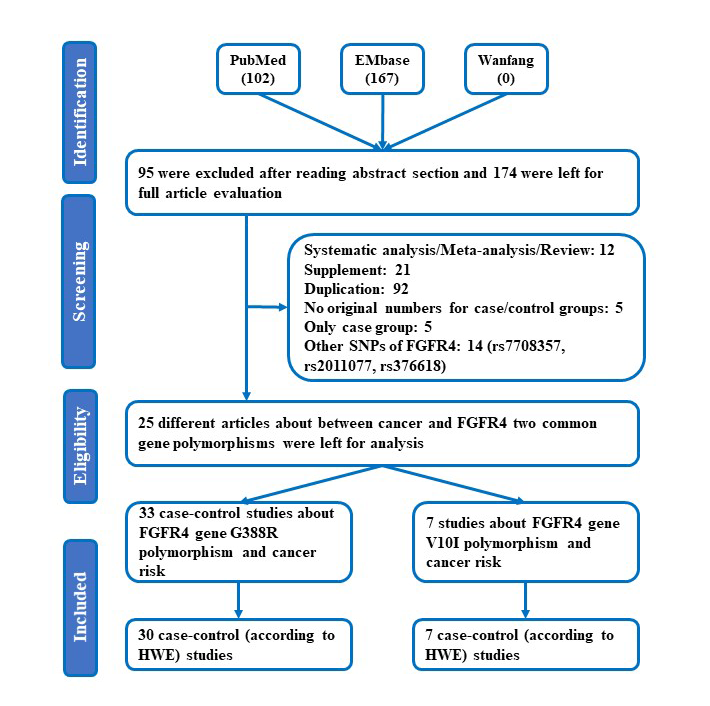

Supplement: Supplementary file 1 — Supplementary Figure. [file 41598_2020_80146_MOESM1_ESM.doc]
